# Kinetics of plasma von Willebrand factor in acute myocardial infarction patients: a meta-analysis

**DOI:** 10.18632/oncotarget.20091

**Published:** 2017-08-09

**Authors:** Xia Wang, Junyu Zhao, Yong Zhang, Xiujuan Xue, Jie Yin, Lin Liao, Cuiping Xu, Yinglong Hou, Suhua Yan, Ju Liu

**Affiliations:** ^1^ Medical Research Center, Shandong Provincial Qianfoshan Hospital, Shandong University, Jinan, 250014 Shandong, China; ^2^ Department of Endocrinology, Shandong Provincial Qianfoshan Hospital, Shandong University, Jinan, 250014 Shandong, China; ^3^ Department of Cardiology, Shandong Provincial Qianfoshan Hospital, Shandong University, Jinan, 250014 Shandong, China; ^4^ Department of Nursing, Shandong Provincial Qianfoshan Hospital, Shandong University, Jinan, 250014 Shandong, China

**Keywords:** von Willebrand factor, kinetics, acute myocardial infarction, meta-analysis

## Abstract

Previous studies have shown a variation in plasma level of von Willebrand factor (vWF) in acute myocardial infarction (AMI) patients but with contentious results. In this study, we performed a meta-analysis to evaluate the kinetics of plasma vWF after AMI. A total of 11 qualified studies were obtained through systematical search in PubMed, Web of science, Cochrane Library database and CNKI, followed by search of reference lists, involving 519 AMI patients and 466 non-AMI controls. The standard mean difference (SMD) and 95% confidence intervals (95% CI) were calculated using random-effects model. Results indicated that the plasma vWF was significantly increased in the first several hours after onset of AMI (SMD = 1.94, 95% CI = 1.39–2.48, *P* < 0.001) and stayed at high level until 24 h (SMD = 1.17, 95% CI = 0.45–1.89, *P* = 0.001). Elevated level of vWF appeared to persist for one week and reduced to normal until the fourteenth day after AMI (SMD = 0.44, 95% CI = −0.14–1.02, *P* = 0.14). Subgroup analysis revealed that the high level of vWF lasted just for 1 day in patients with a symptom duration ≤ 6 h before admission. For patients with a symptom duration > 6 h, elevated vWF was found in all 7 days except day 1. Our findings determined the kinetics of plasma vWF after AMI, and might provide a new insight in monitoring AMI progression.

## INTRODUCTION

Acute myocardial infarction (AMI) is one of leading causes of mortality. In 2008, over 3,000,000 individuals developed ST-elevated myocardial infarction (SETMI) and 4,000,000 developed non-ST-elevated myocardial infarction (non-STEMI) worldwide [1]. AMI is caused by rupture of atherosclerotic plaques, exposure of sub-endothelial procoagulant factors, and subsequent thrombus formation, which leads to the complete or incomplete occlusion of coronary arteries [2]. As the most serious type of acute coronary syndromes (ACS), AMI may lead to cardiac death and/or heart failure [3]. The current diagnosis for AMI is mainly dependent on electrocardiograph (ECG) and circulating myocardial enzymes such as ultrasensitive troponin I or T and creatine kinase MB [4]. To date, no clinical marker is available for the accurate assessment of AMI process [5].

Von Willebrand factor (vWF), a large multimeric plasma glycoprotein, is well known for its role in hemostasis, where it binds to platelets and to the constituents of the sub-endothelial connective tissue [6, 7]. Following synthesis, vWF is transported to storage organelles in both megakaryocytes/platelets (α-granules) and endothelial cells (Weibel-Palade bodies) [8, 9]. Although platelets secrete vWF, circulating vWF level has been shown to depend almost entirely on vWF from endothelial cells [6, 10] through both consecutive and regulated pathways. A large amount of vWF is stored in Weibel-Palade bodies in endothelial cells and released towards the lumen of blood vessels in response to various stimuli [11, 12]. Particularly, vWF is released during endothelial injury and is recognized as a marker of endothelial dysfunction [13, 14].

Previous studies have suggested a crucial role of vWF in thrombus formation at sites of high shear rates by mediating platelets adhesion and aggregation [15–17]. Increased plasma vWF level is considered as one of the risk factors for AMI [18–20]. Furthermore, an elevation in plasma level of vWF has been extensively reported in AMI patients [21–32], but the dynamic changes of vWF concentration during the progression of AMI have been contentious as they can be influenced by many factors. For example, patients with Thrombolysis in Myocardial Infarction (TIMI) grade 3 were reported to have less vWF in plasma than that of other patients [24, 30]. To get a more comprehensive understanding of variations of the vWF levels in AMI patients, we performed a meta-analysis, including 519 AMI patients and 466 healthy or non-ACS controls, to determine the kinetics of plasma vWF after AMI.

## RESULTS

### Search results and characteristics of included studies

**Figure 1:**
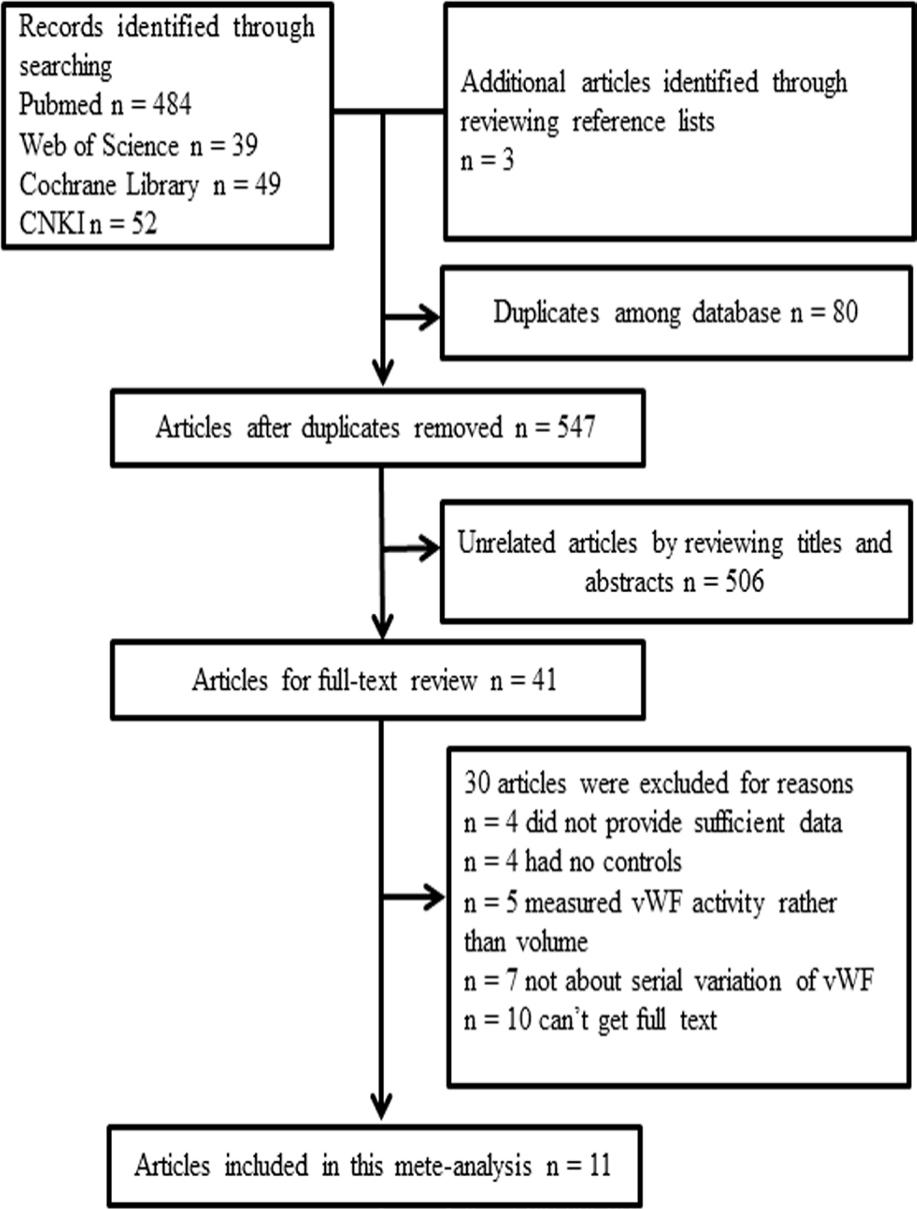
The flow chart of the literature search and selection process

The initial search strategy yielded 627 hits, of which 80 were excluded for duplicated recordings. After carefully reviewing the titles and abstracts, 41 candidate articles were screened out for further full-text reading, and 506 unrelated articles were excluded. In addition, 30 full-text reviewed articles were excluded due to disqualification of inclusion criteria. The flow chart of the entire literature search process is shown in Figure 1. The study of Xie *et al.* [26] was eliminated due to the low quality (5 scores). Patients in study of Zhou *et al.* [24] were classified into two groups according to TIMI grade after reperfusion therapy, and these two groups were independently incorporated in our meta-analysis. As a result, we included 11eligible studies [22–25, 27–32] in this meta- analysis with a total of 519 AMI patients and 466 healthy or non-ACS controls. The included studies provided the plasma level of vWF at different time points after AMI (from on admission to 14 days). The control group included healthy volunteers and patients with a normal coronary angiography. The characteristics of the 11 eligible articles are summarized in Table 1.

**Table 1:** Characteristics of the studies included in the meta-analysis

Year	Author	Age (mean) C/CTL	Gender (male %) C/CTL	Sample size C/CTL	Duration before admission	Treatment modality	Measuring methods	TIMI grade (number)
2016	Teunissen [22]	59/59	78/78	30/30	≦ 6 h	PCI & antiplatelet & anticoagulation	ELISA	3 (57)2 (3)
2015	Regueiro [32]	53.7/54.7	85/84.7	100/98	≦ 24 h	PCI or Thrombolysis	ELISA	NR
2012	Jia [23]	62.64/ 47.45	73/68	48/19	NR	Antiplatelet & anticoagulation	ELISA	NR
2011(1)	Zhou [24]	63.4/58.44	58/60	43/22	NR	PCI	ELISA	3 (43)
2011(2)	Zhou [24]	63.4/58.44	60/60	43/21	NR	PCI	ELISA	≦ 2 (43)
2007	Matsukawa [25]	65/65	73/63	92/40	≦ 24 h	PCI & antiplatelet	ELISA	NR
2000	Sakai [27]	59/50	NR	51/58	≦ 6 h	PCI & antiplatelet & anticoagulation	EIA	NR
2000	Lip [31]	62/62	70/70	17/59	≦ 12 h	Thrombolysis & anticoagulation	ELISA	NR
2000	Xie [26]	68/61	75/50	16/16	NR	PCI or Thrombolysis	EIA	3 (16)
1996	Tousoulis [28]	60/46	94/38	16/8	NR	Antiplatelet & anticoagulation	ELISA	NR
1992	Norris [29]	58/52	81/81	51/36	≦ 6 h	Thrombolysis	EIA	NR
1990	Andreotti [30]	58/58	71/71	12/12	≦ 6 h	Thrombolysis & anticoagulation	ELISA	≧ 2 (16)< 2 (8)

Abbreviations: C/CTL, case/control group; NR, unreported; PCI, percutaneous coronary intervention; ELISA, enzyme-linked immunosorbent assay; EIA, enzyme immunoassay; TIMI, thrombolysis in myocardial infarction.

### Kinetics of plasma vWF

**Figure 2:**
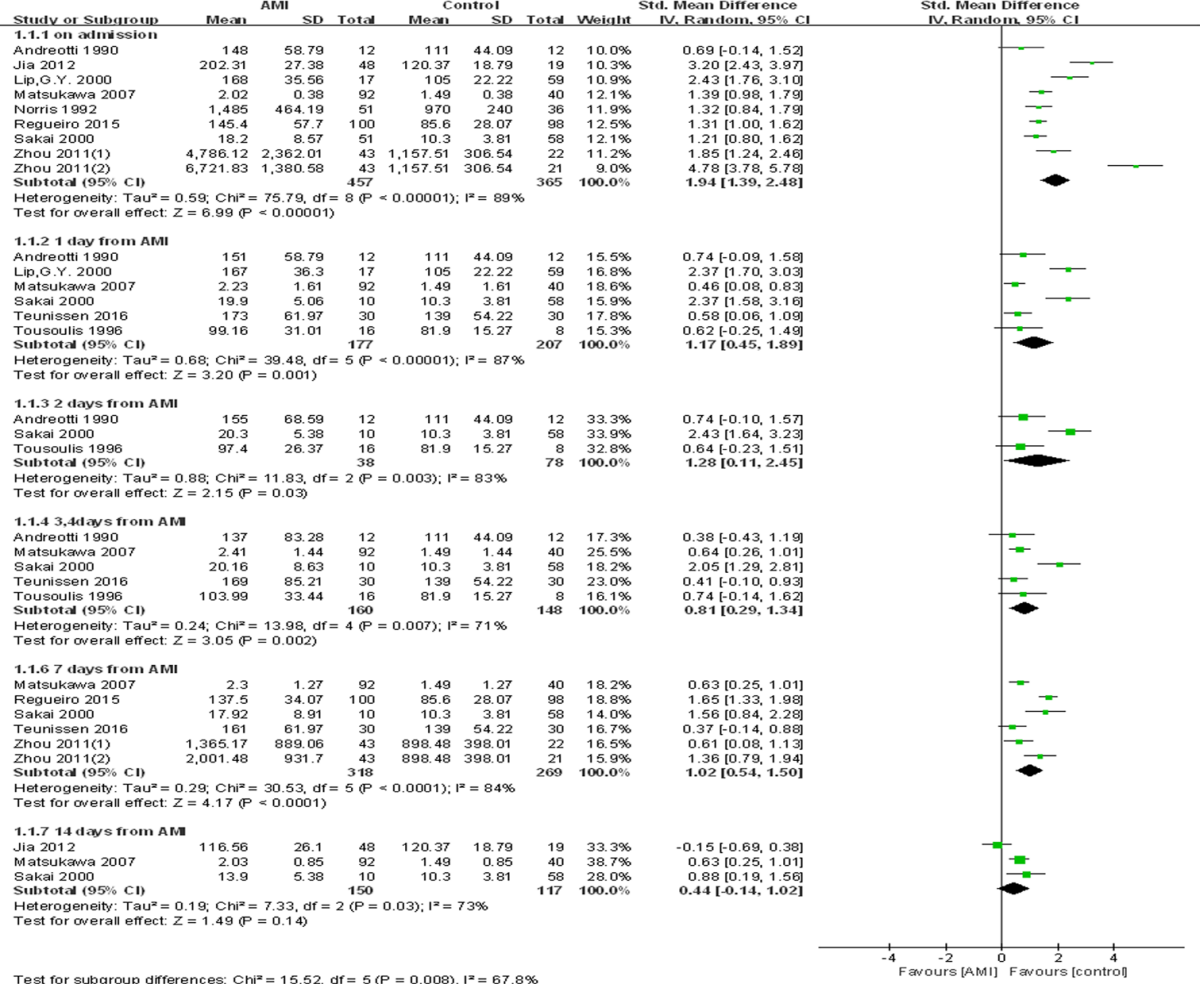
Forest plot of meta-analysis of kinetics in plasma vWF concentration after AMI Each block represents a study and the area of each block is proportional to the weight of that study. The horizontal line represents each study's 95% confidence interval (CI) for the effect. The centre of the diamond is the pooled effect across studies, and the width of the diamond denotes its 95% CI. AMI, acute myocardial infarction; IV, invers variance; SD, standard deviation; Std, standard.

The meta-analysis revealed that plasma level of vWF was significantly higher in AMI patients compared with healthy volunteers or non-ACS patients. The high level of plasma vWF persisted for one week. The pooled SMD was 1.94 (95% CI = 1.39–2.48, *P* < 0.001) on admission, 1.17 (95% CI = 0.45–1.89, *P* = 0.001) and 1.28 (95% CI = 0.11–2.45, *P* = 0.03) on day 1 and day 2, 0.81 (95% CI = 0.29–1.34, *P* = 0.002) on day 3–4, and 1.02 (95% CI = 0.54–1.50, *P* < 0.001) on day 7. However, no statistically significant difference was found on day 14 (SMD = 0.44, 95% CI = −0.14–1.02, *P* = 0.14). It was noted that there was significant heterogeneity across studies (Figure 2). As a result, random-effects model was adapted.

### Quality evaluation

The results of the quality assessment of the included studies are shown in Table 2. Eight studies were determined as high quality [22, 24, 25, 27, 29–32], and two were determined as medium quality [23, 28]. These 11 studies received an average score of 7.18. One report [26] of low quality (5 scores) was excluded from this meta-analysis.

**Table 2:** Quality assessment of the included studies based on the Newcastle–Ottawa Scale

Author	Study design	Selection	Comparability	Outcome	Total scores
Teunissen [22]	Cohort study	⋆⋆⋆	⋆⋆	⋆⋆⋆	8
Regueiro [32]	Cohort study	⋆⋆⋆	⋆⋆	⋆⋆⋆	8
Jia [23]	Cohort study	⋆⋆	⋆	⋆⋆⋆	6
Zhou [24]	Cohort study	⋆⋆⋆	⋆⋆	⋆⋆⋆	8
Matsukawa [25]	Cohort study	⋆⋆⋆	⋆⋆	⋆⋆⋆	8
Sakai [27]	Cohort study	⋆⋆⋆	⋆⋆	⋆⋆	7
Lip [31]	Cohort study	⋆⋆⋆	⋆⋆	⋆⋆⋆	8
Xie [26]	Cohort study	⋆	⋆	⋆⋆⋆	5
Tousoulis [28]	Cohort study	⋆⋆	⋆	⋆⋆⋆	6
Norris [29]	Cohort study	⋆⋆	⋆⋆	⋆⋆⋆	7
Andreotti [30]	Cohort study	⋆⋆⋆	⋆⋆	⋆⋆⋆	8

### Subgroup analysis

Subgroup analyses were conducted in all the groups except the groups of day 2 and 14 after AMI since no sufficient studies was included for these two groups. Results showed that for the AMI patients with a symptom duration ≦ 6 h before admission, significantly elevated level of plasma vWF was identified on admission and on day 1 after AMI (SMD = 1.19, 95% CI = 0.90–1.48, *P* < 0.001 on admission; SMD = 1.21, 95% CI = 0.11–2.31, *P* = 0.03 on day 1), while no significant elevation was found on 3–4 days and 7 days from AMI (SMD = 0.93, 95% CI = −0.10–1.97, *P* = 0.08 on day 3–4; SMD = 0.94, 95% CI = −0.22–2.10, *P* = 0.11 on day 7). For patients with a symptom duration > 6 h or unreported, elevated vWF was found in all 7 days except day 1 (Table 3). In patients treated with percutaneous coronary intervention (PCI), the higher level of vWF persisted for 7 days. However, for those without PCI, significant difference was found only on day 1 while disappeared on day 3 and 4 after therapy. On day 7, the pooled SMD was 0.49 (95% CI = 0.12–0.85, *P* = 0.009) for patients with TIMI grade 3 and 1.29 (95% CI = 0.73–1.84, *P* < 0.001) for the others, suggesting a higher level of vWF was found even under an ideal reflow condition of coronary.

**Table 3:** Summary results of plasma vWF levels in AMI patients and controls

Variables		No. of comparisons	No. of subjects		Meta-analysis			Heterogeneity		Test for subgroup differences	
			AMI	Controls	SMD	95% CI	*P*	*I*^2^ (%)	*P*	*I*^2^ (%)	*P*
Duration before admission											
On admission	≦6 h	3	114	106	1.19	0.90–1.48	< 0.001	0	0.43	87.4	0.005
	> 6 h/NR	6	343	259	2.40	1.61–3.20	< 0.001	92	< 0.001		
1 day	≦6 h	3	52	100	1.21	0.11–2.31	0.03	86	< 0.001	0	0.94
	> 6 h/NR	3	125	107	1.14	−0.10–2.39	0.07	92	< 0.001		
3–4 days	≦6 h	3	52	100	0.93	−0.10–1.97	0.08	85	0.001	0	0.47
	> 6 h/NR	2	108	48	0.65	0.30–1.00	< 0.001	0	0.55		
7 days	≦ 6 h	2	40	88	0.94	−0.22–2.10	0.11	85	0.009	0	0.93
	> 6 h/NR	4	278	181	1.07	0.49–1.65	0.0003	86	< 0.001		
PCI											
1 day	Yes	3	132	128	1.07	0.12–2.03	0.03	90	<0.001	0	0.80
	No	3	45	79	1.27	0.08–2.45	0.04	85	0.001		
3–4 days	Yes	3	132	128	0.97	0.18–1.76	0.02	85	0.001	0	0.39
	No	2	28	20	0.54	−0.05–1.14	0.07	0	0.55		
TIMI grade											
7 days	TIMI = 3	2	73	52	0.49	0.12–0.85	0.009	0	0.53	82.1	0.02
	TIMI≦2/NR	4	245	217	1.29	0.73–1.84	< 0.001	82	< 0.001		

Abbreviations: No, number; AMI, acute myocardial infarction; SMD, standardized mean difference; NR, unreported; PCI, percutaneous coronary intervention; TIMI, thrombolysis in myocardial infarction.

### Sensitivity analysis

Each study was excluded sequentially to evaluate the influence of an individual study on the results. No study fundamentally changed the combined effects at any time points except on day 14. Furthermore, the studies of Sakai *et al*. [27] and Jia *et al*. [23] were found to be the main source of heterogeneity. Elimination of the former study yielded similar results without heterogeneity (on day 2: SMD = 0.69, 95% CI = 0.09–1.29, *P* = 0.02, *I^2^* = 0%; on day 3–4: SMD = 0.55, 95% CI = 0.28–0.83, *P* < 0.001, *I^2^* = 0%). When the later study was eliminated from analysis, a statistically significant difference of plasma vWF was also found on day 14 (SMD = 0.69, 95% CI = 0.36–1.02, *P* < 0.001, *I^2^* = 0%), suggesting that the original result for 14 days after AMI was not stable.

### Publication bias

**Figure 3:**
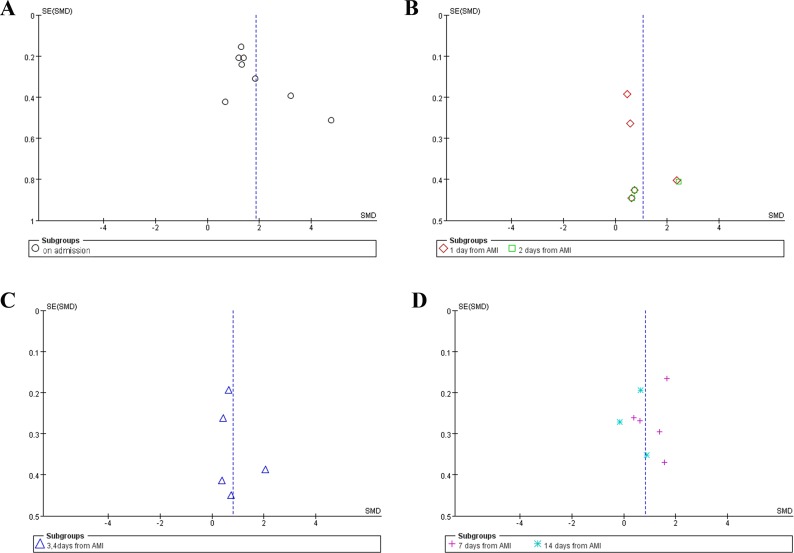
Funnel plot of publication bias No publication bias was pbserved in any groups. (**A**) Funnel plot of studies on admission. (**B**) Funnel plot of studies on day 1 and 2 after AMI. (**C**) Funnel plot of studies on day 3 and 4 after AMI. (**D**) Funnel plot of studies on day 7 and 14 after AMI.

Funnel plot was performed to evaluate the publication bias of literatures. As shown in Figure 3, no significant publication bias was observed.

## DISCUSSION

Over the 11 studies, 519 AMI patients and 466 healthy or non-ACS controls were included in this meta-analysis. The pooled SMD indicated that the concentration of plasma vWF was significantly higher in AMI patients than controls and persisted for one week after reperfusion therapy. Sensitivity analysis showed that the pooled results were robust. However the result for 14 days after AMI was not stable because a reverse change of the pooled SMD was caused by the removal of Jia *et al.* literature. Thus, additional studies are needed to obtain a more reliable assessment of plasma vWF level 14 days after AMI. In addition, for patients with a symptom duration > 6 h or unreported, significantly elevated vWF was found in all 7 days except day 1. The negative result of day 1 may be resulted from unrevealed clinical heterogeneity due to the limited information provided by certain literatures.

vWF is a multimeric protein primarily produced by endothelial cells in circulation [6]. Newly released vWF composes a relatively higher amount of larger multimers which have a crucial role in platelet thrombus formation [33]. After release, the peptide bond within the vWF A2 domain is cleaved by ADAMTS13 which maintains the normal size distribution of vWF multimers [34–36]. Decreased ADAMTS13 and increased vWF multimers were found in AMI patients [22, 25, 26, 37, 38], suggesting that these factors could be biomarkers of AMI progression. vWF secretion can be stimulated by inflammatory cytokines [39] and hypoxia [40]. Our subgroup analysis revealed that symptom duration before admission and TIMI grade after reperfusion therapy also had various effects on vWF level after the onset of AMI. Results indicated that early on admission could prevent further elevation of vWF level. However, PCI, one of the most common surgical procedures for myocardial infarction patients, did not show a significant impact on plasma vWF in AMI patients.

vWF plays a pivotal role in the process of thrombus formation, especially under flow conditions [41]. First, vWF promotes platelet aggregation by binding to the receptors GPIb-IX-V complex and GPIIb/IIIa on the surface of platelet [42]. Second, vWF mediates platelet adhesion through binding to collagens located in the subendothelial connective tissue [43]. Furthermore, vWF acts as a carrier protein for coagulation factor VIII and facilitates fibrin clot formation [2]. AMI is a process involving the formation of thrombus, the injury of endothelial cells and the exposure of subendothelial collagen. Previous studies have found an association between vWF and AMI [22–32]. The protocol for detection of plasma vWF level has been well-established in clinical laboratories. Compared with existing myocardial enzymology, vWF has advantages as an indicator of AMI progression. Myoglobin is a sensitive but not specific biomarker of myocardial injury, as it appears in blood 1–3 h after AMI and returns to normal values after 1–1.5 days [44, 45]. CK-MB mass is only sensitive in 6–72 hours after the onset of AMI [46]. At present, cardiac troponins are well accepted biomarkers for diagnosing myocardial injury for its rapid release and sufficient diagnostic window [47]. Similarly, the plasma vWF elevates rapidly after the onset of AMI and persists at a high level for at least one week after AMI. Although whether vWF plays a causal role in AMI remains unclear, the increase of vWF has prognostic value and might be used as an independent predictor for short-term adverse clinical outcomes [18, 48–50].

Our study has several advantages. First, this study meta-analytically identified the dynamic changes of plasma vWF in AMI patients and its influencing factors. The dramatic increase of plasma vWF implies its potential roles in the early diagnosis of AMI, and its kinetics in plasma levels could serve as an adjunct to the detection of AMI progression. Second, all the studies included in this meta-analysis were medium-to-high quality as assessed by Newcastle-Ottawa Quality Assessment Scale. Third, both sensitivity analysis and publication bias assessment confirmed the robustness and reliability of our results.

However, there were limitations in our study. First, significant heterogeneity was found in this meta-analysis. When the publication leading to major heterogeneity was eliminated, the difference of plasma vWF level was significant at 14 days after AMI, which was opposite to the original result. Second, detailed information regarding symptom duration before admission and TIMI grade were not available in several studies, which were important considerations for subgroup analysis. Moreover, we were unable to determine a causal or consequential role of vWF in progression of AMI.

In conclusion, our meta-analysis confirmed that plasma level of vWF is remarkably elevated in AMI patients compared with healthy or non-ACS controls. The higher level of plasma vWF persists at least for one week, and then gradually reduces to normal levels. This study suggests that the detection of plasma vWF levels may be useful in clinical evaluation of AMI. However, the conclusions should be interpreted with caution due to the limited sample size. Further studies are needed to achieve more comprehensive assessment of kinetics of plasma vWF after AMI.

## MATERIALS AND METHODS

### Search strategy

A systematic review of the literature was performed in PubMed, Web of Science, Cochrane Library database and CNKI online facilities. Studies reporting the variation of plasma vWF in AMI patients and published up to August 2016 were identified and analyzed, with a language restriction of English and Chinese. Both Medical Subject Heading (MeSH) terms and free text terms were used. For example, the search terms for vWF were “von Willebrand Factor”, “Factor VIIIR-Ag”, “Factor VIIIR-RCo”, “Ristocetin-Willebrand Factor”, “von Willebrand Protein”, “Factor VIII-Related Antigen”, “Ristocetin Cofactor”, “Plasma Factor VIII Complex” and “vWF”. The search terms for AMI were “Myocardial Infarction”, “Infarct*, Myocardial”, “Myocardial Infarct*”, “Cardiovascular Stroke*”, “Stroke*, Cardiovascular”, “Heart Attack*” and “AMI”. In addition, relevant reviews and articles on the citation lists were manually searched to identify other potentially eligible studies.

### Study selection and data extraction

Two reviewers independently screened the titles, abstracts, and full texts of selected articles. The inclusion criteria included: (1) studies reporting the dynamic changes of plasma vWF after AMI; (2) specified diagnosis of AMI according to both clinical syndromes and ECG or myocardial enzymes tests; (3) studies written in English or Chinese. Exclusion criteria were settled as follows: (1) studies that were not related to the variation of vWF after AMI; (2) studies conducted on patients who had other severe disease such as von willebrand disease, or that were not on humans; (3) studies lacking complete data or without controls.

The following data were extracted from each eligible study if available: first author's name, publication year, age and sample size, percentage of males, mean and standard deviation (mean ± SD) or mean and standard error (mean ± SE) of plasma vWF concentration, treatment modality, symptom duration before admission, TIMI grade and measuring methods. In two studies where the data were presented in graph format but authors could not be reached, we extracted the data using Engauge Digitizer software (version4.1, free software downloaded from http://sourceforge.net). Furthermore, if the data were presented in median and interquartile range (IQR) format, SMD was calculated according to the formulations recommended by Cochrane Handbooks. All the information was collected by two independent reviewers and any discrepancy was resolved by discussion with each other or by a third author.

### Quality assessment

The methodological quality of the included studies was assessed by two independent authors according to the criteria of Newcastle-Ottawa Quality Assessment scale (NOS) for assessing the quality of nonrandomized and observational studies in meta-analysis. Those scored ≧ 7 were considered as high quality and those scored ≦ 5 as low quality.

### Statistical analysis

All the statistical analyses were performed using Review Manager software (RevMan5.3, Cochrane Collaboration, Oxford, UK, http://community.cochrane.org). We calculated SMD with 95% CI as a measure of pooled effects because different units of measurement were used across studies (%, ng/ml, ug/ml, U/L, U/ml, IU/L). A *P* value < 0.05 was considered statistically significant. All the studies were weighted by an inverse-variance method, thus the studies with larger samples accounted for higher weight. Random-effects model was chosen considering that there were potential heterogeneity across studies resulted from the variability in ethnicity and race of the subjects, differences in disease severity, inconsistent control of confounding factors and other unavailable information. Inter—study heterogeneity was evaluated using the Cochran's Q and Higgins's *I^2^* statistics, and a significant heterogeneity was considered if *P* < 0.1 or *I ^2^* > 50%.

Subsequent subgroup analyses were conducted to identify the source of potential heterogeneity based on the symptom duration before admission (≦ 6 h or longer), treatment modality (PCI or thrombolysis), and TIMI grades (TIMI = 3 or not) after reperfusion therapy. We also performed sensitivity analysis by removing studies one by one to estimate the stability of meta-analysis. In addition, publication bias for each group studies was assessed by visual inspection of funnel plot.

## Abbreviations

vWF: von Willebrand factor
AMI: acute myocardial infarction
SMD: standard mean difference
95% CI: 95% confidence intervals
STMI: ST-elevated myocardial infarction
non-STEMI: non-ST-elevated myocardial infarction
ACS: acute coronary syndromes
ECG: electrocardiograph
TIMI: Thrombolysis in Myocardial Infarction
PCI: percutaneous coronary intervention
SD: standard deviation
SE: standard error
IQR: interquartile range
NOS: Newcastle-Ottawa Quality Assessment Scale
CK-MB: creatine kinase MB
